# Bilateral Cystic Adrenal Neuroblastoma with Cystic Liver metastasis

**DOI:** 10.21699/ajcr.v8i1.517

**Published:** 2017-01-05

**Authors:** Mine Aslan, Deniz Alis, Ayse Ucar Kalyoncu, Hatice Arioz Habibi, Gul Nihal Ozdemir, Basak Koc, Ibrahim Adaletli

**Affiliations:** 1 Department of Radiology, Istanbul University, Cerrahpasa Medical Faculty, İstanbul /Turkey; 2 Department of Pediatric Oncology, Kanuni Sultan Suleyman Training and Research Hospital Istanbul/Turkey

**Keywords:** Bilateral congenital cystic neuroblastoma, Cystic metastasis

## Abstract

Bilateral congenital cystic adrenal neuroblastoma (NB) with cystic liver metastasis is a very rare condition and only few cases have been reported in the literature. Herein we report a case of a congenital bilateral cystic adrenal NB with cystic liver metastasis and briefly discuss characteristic imaging features of cystic NB.

## CASE REPORT

A 3-month-old baby presented to clinic with marked abdominal distention. His medical history was insignificant. Laboratory findings were within normal limits except for mildly elevated serum transaminases (AST: 54 IU/L, ALT: 45 IU/L) and neuron-specific enolase (NSE: 21.9 ng/ml). An abdominal ultrasound (US) was performed. US revealed 10x10x12cm heterogeneous cystic mass in the right suprarenal region, which was displacing the right kidney downward. US scan also showed a 2x2x2cm anechoic cystic mass in the left adrenal gland. Borders between the right lobe of the liver and lesion were indistinguishable. Multiple anechoic cystic lesions up to 10mm were detected in the right and the left lobes of the liver. Intravenous contrast enhanced magnetic resonance imaging (MRI) was performed for further evaluation of the lesions to avoid the ionizing radiation of computed tomography (CT) scan. MRI revealed that both lesions were cystic in nature and thick walls of the lesion were enhanced with contrast. No solid components were detected in lesions. Calcifications were noted in the inferior-posterior part of the right-sided suprarenal mass. Lesions in the liver also showed no contrast enhancement on MRI (Fig.1). Cystic nature of the lesion, mildly increased NSE and pres¬ence of multiple cystic lesions in the liver, were sup¬porting the diagnosis of congenital bilateral adrenal NB with cystic liver metastasis. Bone marrow aspiration was negative for tumor cells and a proba-ble diagnosis of neuroblastoma was made. In the following days, the patient started to develop a sig-nificant shortness of breath due to rapidly growing abdominal masses (NSE also increased) and under-went operation. During the operation, the right-sided lesion was dissected carefully from the surface of the liver and the right kidney. Then both lesions in the adrenal gland were completely excised. Aspira-tion was performed from one of the cystic lesions in the liver for histopathologic examination. The pa-thology specimen revealed stroma poor, poorly dif-ferentiated cystic NB with 10 fold N-myc amplifica-tion, which is favoring bad prognosis according to Turkish pediatric oncology group (TPOG) [1] (Fig.2). Aspiration specimen of the lesions in the liver con-firmed metastasis. 123I-metaio¬dobenzylguanidine scan show-ed no patho¬logical uptake after surgery. Patient received 2-cycles of chemotherapy including vincristine, etoposide and carboplatin, after the op-eration. Clinical and labor¬atory findings were normal after 2 cycles of chemotherapy. Cystic metastasis in the liver significantly regressed on US examination.

**Figure:**
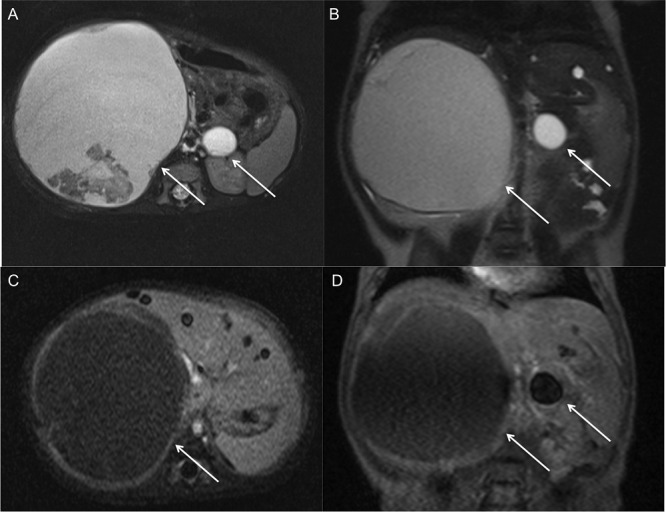
Figure 1: MRI examination of the patient. A, B) Both lesions (arrows) are hyper-intense on axial and coronal T2-weighted sequence. Note the calcification in the postero-inferior part of the right-sided adrenal mass. Liver lesions have the same characteristic as adrenal masses. C, D) Adrenal lesions (Arrows) have thickened walls with contrast enhancement. Note that multiple cystic lesions in the liver do not show contrast enhancement.

**Figure:**
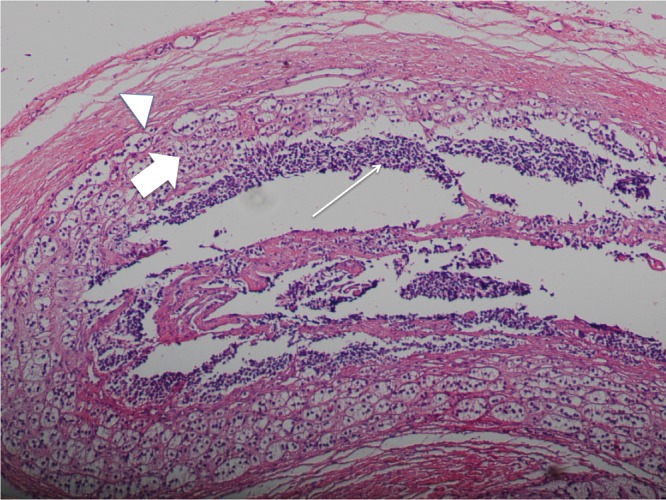
Figure 2: Histology specimen of the cystic adrenal gland neuroblastoma. Tumor cells (arrow), normal cells of the adrenal gland (thick arrow) and fibrous pseudo capsule (arrow head)

## DISCUSSION

NB is the most common extracranial solid tumor among children and commonly diagnosed in the infantile period [2]. Although NB is very common, cystic NB is a rare entity, which is characterized by large cystic lesions that usually originates from adrenal glands [2]. Majority of cases consist of solitary lesions, which are located in left or right adrenal gland. Bilateral involvement of adrenal glands is very rare. Bilateral cystic adrenal NB presenting with multiple cystic liver metastasis is even rarer[3-6].

Diagnosis of the solid form of NB is not complex with the aid of clinical, histopathological, and radiologic findings [7]. Suprarenal masses in infantile period are often presumed to be NB. Although cystic NB is a more commonly encountered entity than adrenal hemorrhage, mesenchymal hamartoma and extra-pulmonary sequestration, these entities still could lead to misinterpretations. Abo-Elenain et al. demonstrated mesechymal hamartoma which might mimic right-sided NB [8]. Haberal et al. identified a case of a right sided-NB, which was misdiagnosed as infantile hemangioendothelioma of the liver that caused severe consumption coagulopathy and bleeding into the adrenal glands [9]. The differential diagnosis of left cystic adrenal NB includes extralobar sequestration, especially in the prenatal period [10]. Differential diagnosis in right-sided masses includes hamartoma and hemorrhage, which more commonly arise diagnostic dilemmas when compared with left sided masses such as sequestration. Bilateral involvement of adrenal glands, cystic metastatic lesions in the liver, thickened walls, calcifications, cystic lesions with solid components and increased color-coding in Doppler US favors the diagnosis of NB [8-10]. Increased serum NSE is the major biochemical finding that supports the diagnosis of cystic NB. Most cystic neuroblastomas have good biological features and show spontaneous regression. This has prompted a “wait and see” strategy, which involves close follow-up with monthly ultrasounds and evaluation of urinary catecholamine levels [11]. However, in our case, respiratory distress of the patient due to rapidly growing mass held us from applying this strategy and we performed operation.

In summary, we present a US and MRI demonstration of a bilateral cystic adrenal gland NB with cystic liver metastasis. We also propound distinct imaging features of cystic neuroblastoma including, irregular, thickened walls, calcification and prominent soft tissue component on gray scale US and increased vascular coding in Doppler US, for differential diagnosis.

## Footnotes

**Source of Support:** Nil

**Conflict of Interest:** None declared

